# Supplementary figures and images for: What are the implications of Zika Virus for infant feeding? A synthesis of qualitative evidence concerning Congenital Zika Syndrome (CZS) and comparable conditions
Source: PLoS Negl Trop Dis. 2020 Oct 21;14(10):e0008731. doi: 10.1371/journal.pntd.0008731 (PMC7605709; doi:10.1371/journal.pntd.0008731)

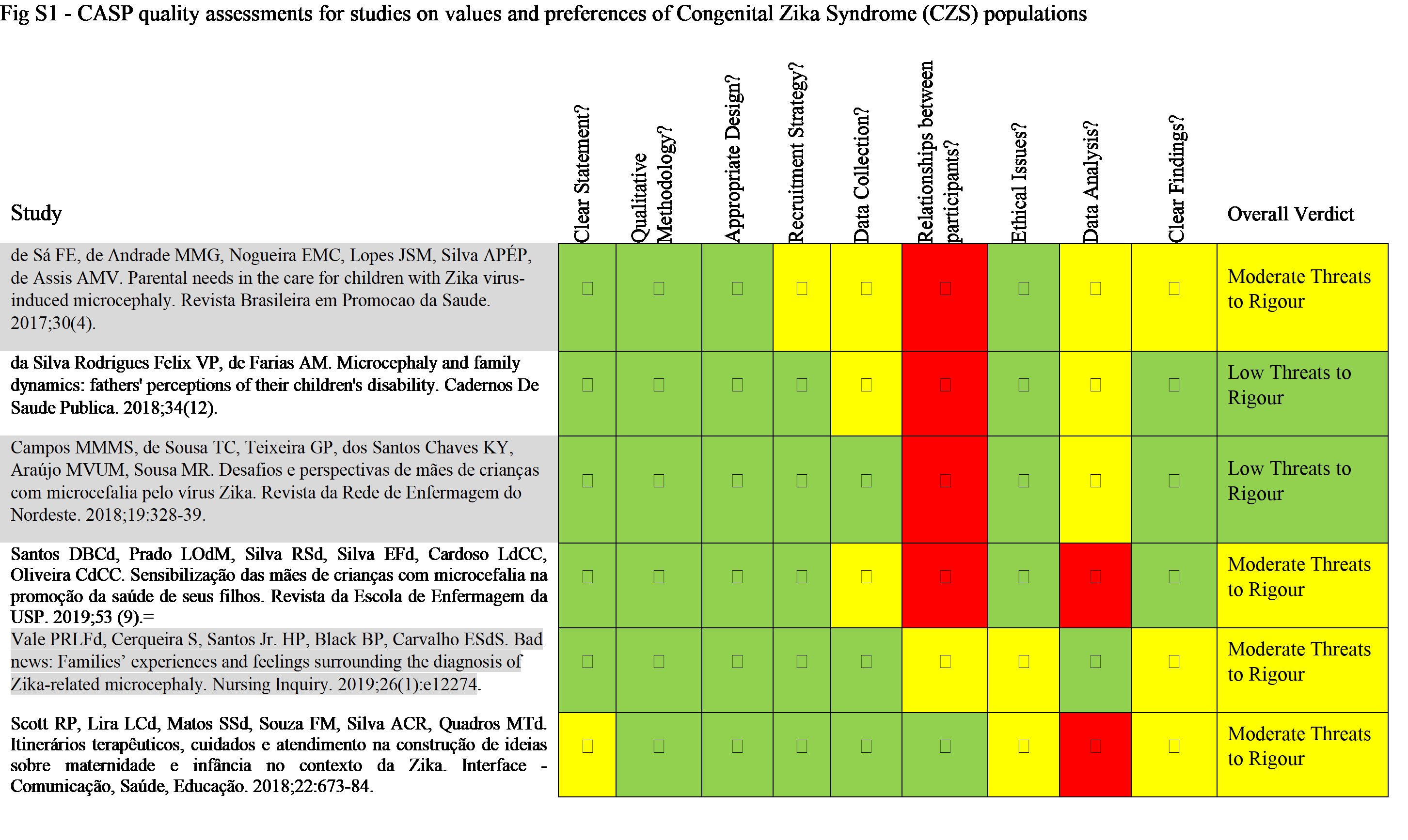

Supplement: S1 Fig — (TIF) [file pntd.0008731.s001.tif]

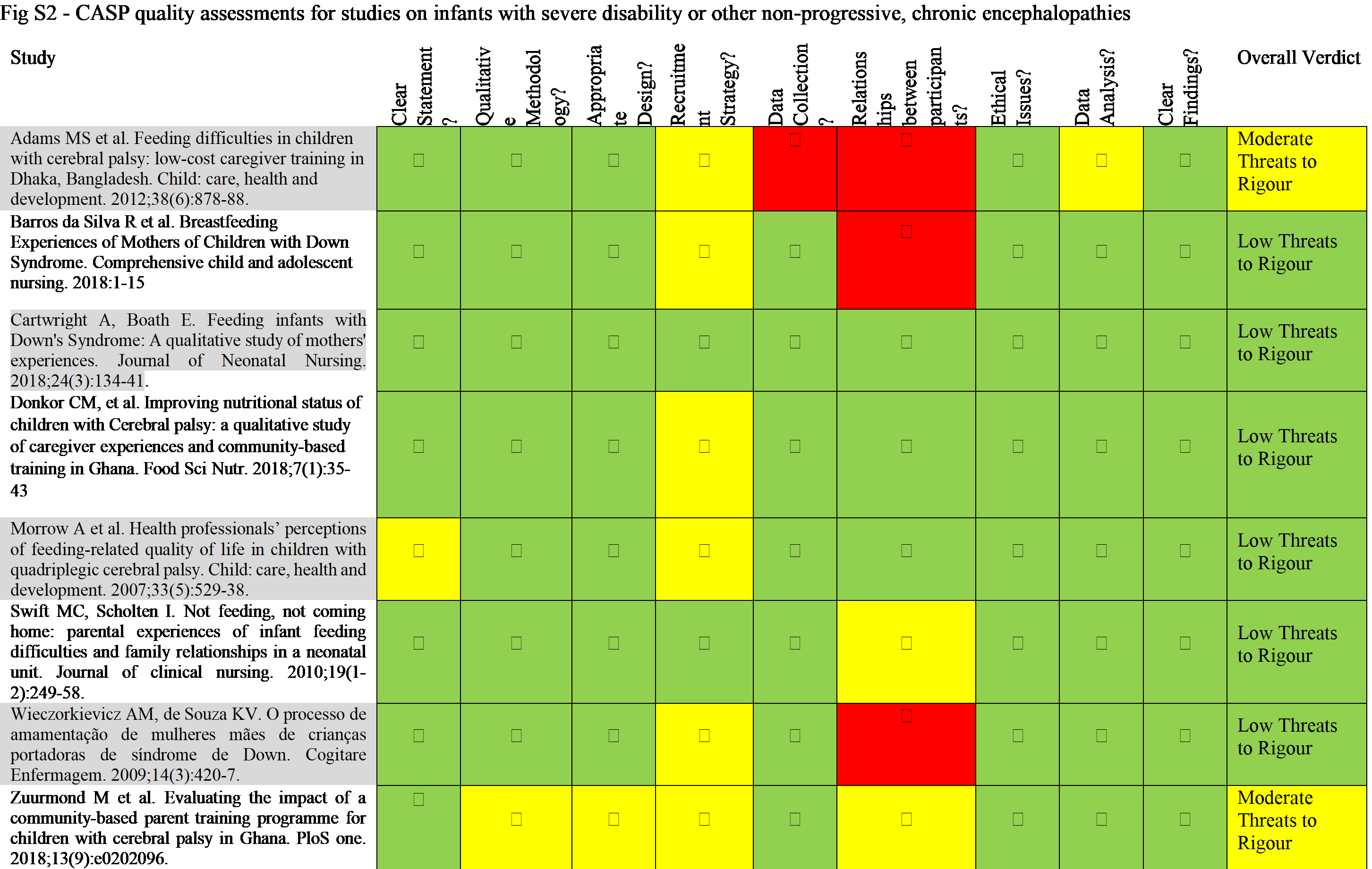

Supplement: S2 Fig — (TIF) [file pntd.0008731.s002.tif]
