# Supplementary material for: What are the implications of Zika Virus for infant feeding? A synthesis of qualitative evidence concerning Congenital Zika Syndrome (CZS) and comparable conditions
Source: PLoS Negl Trop Dis. 2020 Oct 21;14(10):e0008731. doi: 10.1371/journal.pntd.0008731 (PMC7605709; doi:10.1371/journal.pntd.0008731)
Supplement: S1 Table — (DOCX) [file pntd.0008731.s003.docx]

Table S1 - STARLITE specification of each search method

| Element | Zika Virus/ Qualitative Research/ Infant Feeding | Follow up of Included reviews and primary studies | Zika Virus/ Qualitative Research | Infant feeding in the context of severe disability or non-progressive, chronic encephalopathies |
| --- | --- | --- | --- | --- |
| Sampling strategy | Comprehensive | Purposive | Intensity Sampling using brief Qualitative Filter (Qualitative OR Interview(s) or Findings | Purposive (from list of symptoms and conditions) |
| Type of study, | Qualitative Research, Mixed Methods Research | Primary Studies | Qualitative Research | Qualitative research; qualitative component of mixed methods studies |
| Approaches | Subject searching; Citation Searching; Reference Checking | Reference Checking and Citation Searching | Topic Searching, Citation Searching; Google Scholar Searches; Reference Checking | Topic-based searching; follow up of citations |
| Range of Years | 2000-2019 | | | |
| Limits | No language restrictions | | | |
| Inclusions and Exclusions | Exclusions: Surveys | As Above | Exclude: Surveys | Include: Infants  Exclude: Young children |
| Terms Used | See Appendix | Mentions of Zika (or other Transmissible Agents) | See Appendix | For individual symptoms (e.g. swallowing dysphagia, microencephaly) and individual conditions (e.g. Guillain Barre syndrome, Downs syndrome) |
| Electronic sources | - CINAHL (Ovid) - MEDLINE (Ovid) - EMBASE - PsycINFO (Ovid); - Social Science Citation Index (Web of Science); - POPLINE; - LILACS - African Journals Online | Publish or Perish Software | - PubMed - CINAHL - Web of Science - SCIELO - Scopus - LILACS - BIREME - African Journals Online - African Index Medicus - Google Scholar | PubMed; CINAHL (Ovid); Web of Science; Scopus; EMBASE; Google Scholar |
