# Supplementary material for: What are the implications of Zika Virus for infant feeding? A synthesis of qualitative evidence concerning Congenital Zika Syndrome (CZS) and comparable conditions
Source: PLoS Negl Trop Dis. 2020 Oct 21;14(10):e0008731. doi: 10.1371/journal.pntd.0008731 (PMC7605709; doi:10.1371/journal.pntd.0008731)
Supplement: S2 Table — (DOCX) [file pntd.0008731.s004.docx]

Table S2 - Principal Search Strategy

| Zika AND ((explode Infant Nutritional Physiological Phenomena/ OR Bottle Feeding – OR Breast Feeding OR Breast Milk Expression OR Breast Milk OR Milk, Human OR Lactation/ OR Infant Formula/ OR Feeding Behavior/ OR Infant Nutrition OR Infant Feeding) AND (Attitude to Health/ OR Attitude of Health Personnel/ OR Professional-Patient Relations/ OR Patient Education as Topic/ OR Health Knowledge, Attitudes, Practice/ OR Patient Acceptance of Health Care/ OR Qualitative Research/ OR Focus Groups/ OR Interviews as Topic/) OR (Breast Feeding/psychology OR Bottle Feeding/psychology)) |
| --- |
