# Supplementary material for: What are the implications of Zika Virus for infant feeding? A synthesis of qualitative evidence concerning Congenital Zika Syndrome (CZS) and comparable conditions
Source: PLoS Negl Trop Dis. 2020 Oct 21;14(10):e0008731. doi: 10.1371/journal.pntd.0008731 (PMC7605709; doi:10.1371/journal.pntd.0008731)
Supplement: S3 Table — (DOCX) [file pntd.0008731.s005.docx]

Table S3 - Themes and illustrative quotations: Feeding infants with Congenital Zika Syndrome

| Concept | Themes | Illustrative quotations |
| --- | --- | --- |
| Parental anxiety and stress | Parents report uncertainty about how best to feed their child | - Parents in one Zika study reported a “need for guidance on infant feeding, especially on the management of breastfeeding”. [1]. - “Every month, when I go to the doctor’s office, the doctors guide me even more. They talk more about food... hygiene, about a lot of things [participant 1]. Yes, like on the physiotherapy care for us not to lose it, on food, they cannot eat everything [participant 2].”[2]. |
|  | Parents report fear and anxiety due to choking or difficulty swallowing | - “It’s me … giving him food, [solid food]… he chokes with everything … So, in my day to day, the worst thing is this, and giving him water because he chokes” [2]. - “On food, ‘they cannot eat everything’ yet ‘my son eats everything, he just does not eat stone because he does not know how to chew it’ (laughs)” [2]. |
|  | Parents report the burden of feeding can be time-consuming and stressful for mothers | - “It’s me … giving him food, [solid food]… he chokes with everything … So, in my day to day, the worst thing is this, and giving him water because he chokes” [2]. |
| Support | Training | - Mothers asked questions about “difficulties related to breastfeeding and the introduction of first foods, comfort, sleeping position, psychomotor development and care in the home environment” [3]. |

References:

1. de Sá FE, de Andrade MMG, Nogueira EMC, Lopes JSM, Silva APÉP, de Assis AMV. Parental needs in the care for children with Zika virus-induced microcephaly. Revista Brasileira em Promocao da Saude. 2017;30(4).
2. Campos MMMS, de Sousa TC, Teixeira GP, dos Santos Chaves KY, Araújo MVUM, Sousa MR. Desafios e perspectivas de mães de crianças com microcefalia pelo vírus Zika. Revista da Rede de Enfermagem do Nordeste. 2018;19:328-39.
3. Santos DBCd, Prado LOdM, Silva RSd, Silva EFd, Cardoso LdCC, Oliveira CdCC. Sensibilização das mães de crianças com microcefalia na promoção da saúde de seus filhos. Revista da Escola de Enfermagem da USP. 2019;53.
