# Supplementary material for: What are the implications of Zika Virus for infant feeding? A synthesis of qualitative evidence concerning Congenital Zika Syndrome (CZS) and comparable conditions
Source: PLoS Negl Trop Dis. 2020 Oct 21;14(10):e0008731. doi: 10.1371/journal.pntd.0008731 (PMC7605709; doi:10.1371/journal.pntd.0008731)
Supplement: S4 Table — (DOCX) [file pntd.0008731.s006.docx]

Table S4 - GRADE-CERQual Evidence profile: Feeding infants with Congenital Zika Syndrome

| **Summary of review finding** | **Studies contributing to the review finding** | **Methodological limitations** | **Coherence** | **Adequacy** | **Relevance** | **GRADE-CERQual assessment of confidence in the evidence** | **Explanation of GRADE-CERQual assessment** |
| --- | --- | --- | --- | --- | --- | --- | --- |
| Parents report that they often do not know how best to feed their child with microcephaly because the child frequently chokes and has difficulty swallowing | [1-6] | Six studies with moderate or serious methodological limitations because of the lack of reflexivity in four studies, limited details on data analysis in four studies, and a lack of clarity concerning findings in three studies. | Minor concerns about coherence because the link between the data and the finding is clear. Contrasting findings within studies about level of knowledge and provision of information to parents on feeding and food. | Minor to moderate concerns about adequacy given a moderate amount of data, | Minor concerns about relevance because the setting and currency of the studies is highly relevant, although with only six studies the review question is not a focus of these studies. | Moderate confidence | Six studies with minor or moderate concerns about coherence, adequacy, relevance and methodological limitations (only six studies, but all are recent and conducted in the region in Brazil that is principal location of CZS). |
| Parents and others report that feeding a child with swallowing difficulties makes them stressed and anxious, even if they possess information on how to manage this: they fear that they might be doing or do something wrong, and that they might suffocate the child | [1,3-6] | Four studies with moderate or serious methodological limitations because of their lack of reflexivity, limited details on data analysis, and a lack of clarity concerning the findings. | Minor concerns about coherence because the link between the data and the finding is clear. | Moderate concerns about adequacy given a small amount of data. | Moderate concerns about relevance because the setting and currency of the studies is relevant, but there are only three studies and the review question is not a focus of these studies. | Low confidence | Four studies with minor concerns about coherence because the link between the data and findings is very clear, but moderate concerns about methodological limitations, adequacy and relevance (all three studies are very recent and conducted in the one region in Brazil that is the principal location of CZS). |
| Mothers report that problems with feeding can affect bonding with their child | [6] | One study with serious concerns about reflexivity and data analysis, and moderate concerns about data collection. | Serious or moderate concerns about coherence because link between data and finding is inferred. | Serious concerns about adequacy because there is only a small amount of data. | Serious concerns about relevance because the review question is not a principal focus of the study. | Very Low confidence | One study with serious or moderate concerns about methodological limitations, coherence, adequacy and relevance. |
| Mothers report that the burden of feeding, which can be time-consuming and stressful, falls on them | [3] | One study with serious concerns about reflexivity and moderate concerns about data analysis. | Serious or moderate concerns about coherence because link between data and finding is inferred. | Serious concerns about adequacy because there is only a small amount of data. | Serious concerns about relevance because the review question is not a principal focus of the study. | Very Low confidence | One study with serious or moderate concerns about methodological limitations, coherence, adequacy and relevance. |
| Parents feel that the information provided to them by health professionals is mostly inadequate | [3,6] | Two studies with serious or moderate concerns about reflexivity and data collection and analysis | Serious or moderate concerns about coherence because link between data and finding is inferred. | Serious concerns about adequacy because there is only a small amount of data. | Serious concerns about relevance because the review question is not a principal focus of the study. | Very Low confidence | Two studies with serious or moderate concerns about methodological limitations, coherence, adequacy and relevance. |
| Families value training where given | [5,6] | Two studies with serious methodological concerns about the data analysis. One with moderate limitations on the question, ethics and clarity of the findings. The other incompletely describes data collection. | Serious concerns about coherence because link between data and finding is inferred. The other study refers to a subgroup of comparatively well-off mothers with insurance cover. | Serious concerns about adequacy given only a small amount of data. | Moderate concerns about relevance because the review question is not a focus of one study. | Very Low confidence | Two studies with serious or moderate concerns about methodological limitations, coherence, adequacy and relevance. |
| Families experience economic pressures because of the need to buy special food | [5,6] | Two studies with serious methodological concerns about the data analysis. One with moderate limitations on the question, ethics and clarity of the findings. The other incompletely describes data collection. | Serious concerns about coherence because link between data and finding is inferred. The other study refers to a subgroup of comparatively well-off mothers with insurance cover. | Serious concerns about adequacy given only a small amount of data. | Moderate concerns about relevance because the review question is not a focus of one study. | Very Low confidence | Two studies with serious or moderate concerns about methodological limitations, coherence, adequacy and relevance. |

CZS: Congenital Zika Syndrome

References:

1. de Sá FE, de Andrade MMG, Nogueira EMC, Lopes JSM, Silva APÉP, de Assis AMV. Parental needs in the care for children with Zika virus-induced microcephaly. Revista Brasileira em Promocao da Saude. 2017;30(4).
2. Vale PRLFd, Cerqueira S, Santos Jr. HP, Black BP, Carvalho ESdS. Bad news: Families’ experiences and feelings surrounding the diagnosis of Zika-related microcephaly. Nursing Inquiry. 2019;26(1):e12274.
3. Campos MMMS, de Sousa TC, Teixeira GP, dos Santos Chaves KY, Araújo MVUM, Sousa MR. Desafios e perspectivas de mães de crianças com microcefalia pelo vírus Zika. Revista da Rede de Enfermagem do Nordeste. 2018;19:328-39.
4. da Silva Rodrigues Felix VP, de Farias AM. Microcephaly and family dynamics: fathers' perceptions of their children's disability. Cadernos De Saude Publica. 2018;34(12).
5. Scott RP, Lira LCd, Matos SSd, Souza FM, Silva ACR, Quadros MTd. Itinerários terapêuticos, cuidados e atendimento na construção de ideias sobre maternidade e infância no contexto da Zika. Interface - Comunicação, Saúde, Educação. 2018;22:673-84.
6. Santos DBCd, Prado LOdM, Silva RSd, Silva EFd, Cardoso LdCC, Oliveira CdCC. Sensibilização das mães de crianças com microcefalia na promoção da saúde de seus filhos. Revista da Escola de Enfermagem da USP. 2019;53 (9).
