# Supplementary material for: What are the implications of Zika Virus for infant feeding? A synthesis of qualitative evidence concerning Congenital Zika Syndrome (CZS) and comparable conditions
Source: PLoS Negl Trop Dis. 2020 Oct 21;14(10):e0008731. doi: 10.1371/journal.pntd.0008731 (PMC7605709; doi:10.1371/journal.pntd.0008731)
Supplement: S5 Table — (DOCX) [file pntd.0008731.s007.docx]

Table S5 - Themes and illustrative quotations: Feeding in infants with severe disability or nonprogressive, chronic encephalopathies

| Concept | Themes | Illustrative quotations |
| --- | --- | --- |
| Parental anxiety and tress | Parents report uncertainty about how best to feed their child | “She was more sleepy, more floppy, should I be waking her, how often should I be feeding her?” (Hayley)[1]  “I was still adamant that I wanted to breastfeed him, but he was on a very calorie controlled diet so it was hard to be able to do the two. But he was also on a very fluid restricted diet. So I still don't know now whether I did the right thing or whether I made his heart failure worse by persisting and trying as much as I could to allow him to have the experience and practice of sucking, whilst trying to minimise the amount of milk that he got from breastfeeding.” (Jo) [1]  “The internal struggle of like, is it the right thing to do to breastfeed him, because of all the benefits, or is it cruel to make him work for it and to tire him out?” (Rachel) [1]. |
|  | Parents report the burden of feeding can be time-consuming and stressful for mothers | “Since it takes such a long time to feed him, I often do not have the time to do other things. I am unable to leave him and even go and work because of his condition. I need to work and support him and the other children but I just cannot”.[2]  “It is also hard and tiring feeding him. Sometimes, he is messy and I get covered with his food. When he is being fed, he spurts out the food and some lands on my face. It is very tiresome feeding him” [2]. |
|  | Bonding concerns when feeding is problematic | “Nevertheless, breastfeeding is something that we feel it, that is ours, is that bond that no one else can have. It is difficult to express with words but that is a sense of completion, fullness, to be a mother, which with breastfeeding is achieve. It is the bond. And the truth is that it is tiring, if not there isn’t some support around . . . it is much more difficult to breastfeed, it is much more practical to pick up the formula and give it.” (Opal) [3]. |
| Health Professionals | Parents feel that the information and support provided to them by health professionals is mostly inadequate | ‘“ … you don't know what you're supposed to be provided with, and most of the time my midwives and health visitors didn't know either’ [2]  “You’d go to a different nurse and they’d have different ways of doing things” [Jane, Int.]; “You’d be that confused” [Penny, Int.,]. “There were too many nurses with too many different ways to try to get you to do it… it just got really frustrating.” [Tricia, Int.][4].  “We did have the breastfeeding support woman come out and she was very lovely, and she did say ‘Oh, I can see he's latched on really well, but not really sucking’ but she offered no solutions.” (Lily) and, “Despite the encouragement to express, Steph recounted that she was given no practical support with this”[2]. “Health professionals who think: Oh, that child has DS and cannot suckle, we will continue to feed with the nasogastric tube, and it was what they said. I picked a poor doctor, also nurses, who felt he had no conditions to breast feed because he had DS”. (Emerald) [1].  “I had support from my brother, the church and the maternity professionals, wow they helped too much, the nurse then, don't even talk! [was fundamental]” [...] (M3) [5].  “I mean we've been really well supported I'd say sort of nutritionally, but perhaps not so much on the actual breastfeeding if that make sense” (Rachel)[2].  “Maternity nurses treated him a lot well, with a lot of affection [...]. They helped, lift his little head when he was suckling [...]” [5]. |
|  | Parents report feeling they have to seek information themselves | “And I was constantly seeking advice from the health professionals, from my midwife and then later from the health visitor, and then she'd say they just don't have the access to any information” [3]  “In a website, Down movement, a Brazilian website who by the way, is a website that I think it has a lot. Very good, very good, I read a lot there, I took the exercises to do with it, loved it. Regarding the DS I go to the internet, because at the Health Centre, they don’t help, they’re not able to do so”. (Agate)[6].  “I think there should be awareness campaigns, more training for nurses, for doctors themselves . . . it takes a lot of training, I think is very important in university, even get practical cases into university, when people are learning, they should be required to attend such training (. . .) They should learn how to deal with parents who are caught by surprise, how to deal with babies who have some kind of disability, and all these things that must be worked on” (Emerald) [6]. |
|  | Parents report a general lack of control | “She doesn’t feel like she belongs to you. You feel like she belongs to them and you’re just visiting’’. [7].  “And … it was really important that there wasn't that separation unless there was an indication that [child’s name] was poorly and needed to go for a specific reason”[3].  “It gave me an opportunity to get involved. So in some ways it was good. Well for me it was good. That’s a bit selfish (laughter). Because … I reckon the first time I fed her with the bottle was really good. That’s pretty important. Yeah, it must’ve made an impression”. [Mark, Int.][1]. |
|  | Parents report that the infant’s weight gain can be the overwhelming focus both for them and for health professionals | “Well, the grams. I mean if all it comes down to is, they will be discharged when they’re putting on weight, then obviously to me, putting on weight is more important than breastfeeding. Because if they said ‘your baby will be discharged when she is breastfeeding’, then that’s completely different. But if it just comes down to the grams on the measure, then who cares how they get it?” [Tricia, Int.]; [1].  ‘“Feeding was everything that was all I could think about, all I could focus on, because I knew how much of a struggle we were both having. And I knew that that was the only way he was going to survive, but also, he needed to gain weight so he could have his surgery”(Jo)” [3].  ‘“Like if they hadn't panicked me and panicked themselves about the weight gain then I probably would have breastfed”’ and “I had the breastfeeding consultant coming over and a little bit of help with the breastfeeding but generally there was more about getting his weight up and using the formula” (Hazel) [3]. |
| Support | Training | “I feel good because my child has stopped vomiting when he eats because you advised me to sit my child up for feeding. And now he has less fever as a result and I feel good”[5].  “When he tilts the head, I help him to bring it forward because [I’ve] been told that if I don’t do that, the food will not go down well“[2]  “At first he could only eat porridge and rice, but now he's able to eat all types of food. I have to modify some solids and for other foods like ampesi [boiled yam], I have to mash it a little bit before I give” [2].  “Because of him [her son] I can't even work, and his father has rejected us. My family members too are not taking care of me. And I have so many problems in the world and it is very hard and difficult.”[4].  “Before, I could not go to any place and leave the child behind. Anytime I went out, it was difficult for my mother to feed him. He used to vomit everything you feed him, but after the training, I also educated my mother and anywhere I go I come back to see him fed well”. (Single mum living with her own mother, son of 2 yrs)[4].  “They are more interested in her and take her on their lap more because they see she is less sick now and more lively because of the training.” [5].  “Through the training we’ve understood what the condition is… we’ve learnt we need to be patient when giving [our children] the food, and also the types of food to give them… We’ve been able to apply [our] knowledge and things are much better’ [2]. |
|  | Resource considerations | “Fathers were almost completely absent from the household, whether because of separation or divorce, working away from home, and infrequent home visits”[4]. |

References:

1. Swift MC, Scholten I. Not feeding, not coming home: parental experiences of infant feeding difficulties and family relationships in a neonatal unit. Journal of clinical nursing. 2010;19(1-2):249-58.
2. Donkor CM, Lee J, Lelijveld N, Adams M, Baltussen MM, Nyante GG, et al. Improving nutritional status of children with Cerebral palsy: a qualitative study of caregiver experiences and community-based training in Ghana. Food Sci Nutr. 2018;7(1):35-43.
3. Cartwright A, Boath E. Feeding infants with Down's Syndrome: A qualitative study of mothers' experiences. Journal of Neonatal Nursing. 2018;24(3):134-41.
4. Zuurmond M, O'Banion D, Gladstone M, Carsamar S, Kerac M, Baltussen M, et al. Evaluating the impact of a community-based parent training programme for children with cerebral palsy in Ghana. PloS one. 2018;13(9):e0202096.
5. Adams MS, Khan N, Begum S, Wirz S, Hesketh T, Pring T. Feeding difficulties in children with cerebral palsy: low‐cost caregiver training in Dhaka, Bangladesh. Child: care, health and development. 2012;38(6):878-88.
6. Barros da Silva R, Barbieri-Figueiredo MdC, Van Riper M. Breastfeeding Experiences of Mothers of Children with Down Syndrome. Comprehensive child and adolescent nursing. 2018:1-15.
7. Wieczorkievicz AM, de Souza KV. O processo de amamentação de mulheres mães de crianças portadoras de síndrome de Down. Cogitare Enfermagem. 2009;14(3):420-7.
