# Supplementary material for: What are the implications of Zika Virus for infant feeding? A synthesis of qualitative evidence concerning Congenital Zika Syndrome (CZS) and comparable conditions
Source: PLoS Negl Trop Dis. 2020 Oct 21;14(10):e0008731. doi: 10.1371/journal.pntd.0008731 (PMC7605709; doi:10.1371/journal.pntd.0008731)
Supplement: S6 Table — (DOCX) [file pntd.0008731.s008.docx]

Table S6 – GRADE-CERQual Evidence profile: Feeding in infants with severe disability or nonprogressive, chronic encephalopathies

| Summary of review finding | Studies contributing to the review finding | Methodological limitations | Coherence | Adequacy | Relevance | GRADE-CERQual assessment of confidence in the evidence | Explanation of GRADE-CERQual assessment |
| --- | --- | --- | --- | --- | --- | --- | --- |
| Parents report that they often do not know how best to feed their child | [1, 2] | Two studies with minor methodological limitations | Minor concerns about coherence because the link between the data and the finding is clear. | Serious concerns about adequacy because there is a substantial amount of rich data, but only two studies | Serious concerns about relevance because the studies focus on the issue, but only in high- resource settings. | Moderate confidence | Two studies with minor concerns about coherence and methodological limitations. The data are rich, but there are serious concerns about adequacy (only two studies) and relevance (only from high- resource settings] |
| Parents report that feeding an infant who has difficulty feeding can be time-consuming and demanding | [2-5] | Three studies with minor methodological limitations, one study with moderate concerns about recruitment and serious concerns about reflexivity, one study with major concerns about data collection and reflexivity. | Minor concerns about coherence because the link between the data and the finding is clear. | Minor concerns about adequacy with a substantial amount of rich data from five studies. | Minor concerns about relevance because the studies focus on the issue, and cover both high and middle/low income settings. | Moderate confidence | Five studies with minor or moderate concerns about coherence and methodological limitations: four studies are of high quality and the data from all studies are rich. Minor concerns about adequacy because there are five studies, and about relevance because these studies were with two exceptions (Ghana, Brazil) conducted in high income settings (UK, Australia]. |
| Parents experience frustration, stress and bonding concerns when feeding their child is problematic | [2-5] | Two studies with minor methodological limitations, and four studies with moderate concerns about recruitment and serious concerns about reflexivity. | Minor concerns about coherence because the link between the data and the finding is clear. | Minor concerns about adequacy because there is a substantial amount of rich data, and contrasting findings within and across studies | Minor concerns about relevance because the studies focus on the issue, across diverse socio-economic settings. | Moderate confidence | Six studies with minor concerns about coherence, and minor or moderate methodological limitations. The data are rich. Minor concerns about adequacy with six studies, and about relevance because studies were conducted across diverse socio-economic settings. |
| Parents feel that the information provided to them by health professionals is mostly inadequate;  Parents feel that the support provided to them by health professionals is mostly inadequate | [1-4,6] | Three studies with minor methodological limitations, and two studies with moderate concerns about recruitment and serious concerns about reflexivity. | Minor concerns about coherence because the link between the data and the finding is clear. | Minor concerns about adequacy because there is a substantial amount of rich data, and there are contrasting findings within and across studies. | Moderate concerns about relevance because the studies focus on the issue, but the socio-economic settings of the studies is mostly limited. | Moderate confidence | Five studies with minor concerns about coherence and minor or moderate methodological limitations: the studies are high quality and the data are rich. Minor concerns about adequacy with five studies, and moderate concerns about relevance because these studies were mostly conducted in a particular socio-economic context (UK, Australia, Portugal, Ghana). |
| Parents report feeling they have to seek information themselves | [1,6] | One study with minor methodological limitations, and one study with moderate concerns about recruitment and serious concerns about reflexivity. | Minor concerns about coherence because the link between the data and the finding is clear. | Moderate concerns about adequacy because there are some rich data, but these are only from three studies. | Moderate concerns about relevance because the studies focus on the issue, but are from limited soci-economic settings [UK, Portugal]. | Low confidence | Two studies with minor concerns about coherence; and minor or moderate methodological limitations: the studies are high quality and the data are rich. There are moderate concerns about adequacy and relevance because there are only two studies from limited settings. |
| Parents report a general lack of control | [1-3,6] | Three studies with minor methodological limitations, and two studies with moderate concerns about recruitment and serious concerns about reflexivity. | Minor concerns about coherence because the link between the data and the finding is clear. | Moderate concerns about adequacy because there is a substantial amount of rich data, and there are contrasting findings within and across studies. | Minor concerns about relevance because the studies focus on the issue, and are from across multiple settings [Australia, Brazil, UK, Portugal]. | Moderate confidence | Four studies with minor concerns about coherence and minor or moderate methodological limitations: the studies are high quality and the data are rich. Moderate concerns about adequacy because there is only five studies, but minor concerns about relevance because they are from across multiple settings (Australia, Brazil, UK, Portugal). |
| Infant’s weight gain can be the overwhelming focus both for them and for health professionals | [1, 2, 5] | Two studies with minor methodological limitations and one with major methodological limitations. | Minor concerns about coherence because the link between the data and the finding is clear. | Moderate concerns about adequacy because there is a substantial amount of rich data, but there are only three studies. | Moderate concerns about relevance because the studies focus on the issue, but the socio-economic setting of the studies is limited. | Moderate confidence | Three studies with minor concerns about coherence and methodological limitations: the studies are mainly high quality and the data are rich. Moderate concerns about adequacy because there are three studies, and about relevance because these studies were conducted in a particular socio-economic context (UK, Australia). |
| Training can alleviate concerns with choking and positioning may avoid risk of vomiting | [2,4,5,7,8] | Three studies with minor methodological limitations, and two studies with moderate concerns about recruitment and serious concerns about reflexivity. | Minor concerns about coherence because the link between the data and the finding is clear. | Minor concerns about adequacy because there is a substantial amount of rich data | Minor concerns about relevance because the studies focus on the issue, across diverse socio-economic settings. | Moderate confidence | Five studies with minor concerns about coherence, and minor or moderate methodological limitations. The data are rich. Minor concerns about adequacy with seven studies, and about relevance because studies were conducted across diverse socio-economic settings. |
| In situations of poverty, feeding problems are exacerbated by lack of resources to buy nutritious food, limited time and facilities for cooking special recipes and lack of access to rehabilitation and health services. Mothers may lack welfare financial assistance or support from the fathers. | [4,5,7,8] | Three studies with minor methodological limitations and one with major methodological limitations | Minor concerns about coherence because the link between the data and the finding is clear. | Moderate concerns about adequacy because there is a substantial amount of rich data, but there are only four studies. | Minor concerns about relevance because the studies focus on the issue, resource issues are more acute in low-resource contexts. | Moderate confidence | Four studies with minor concerns about coherence and minor or moderate methodological limitations: three studies are high quality and the data in all studies are rich. Moderate concerns about adequacy given there are only four studies. Relevance has only minor concerns with three studies from a low-resource socio-economic context |

References:

1. Cartwright A, Boath E. Feeding infants with Down's Syndrome: A qualitative study of mothers' experiences. Journal of Neonatal Nursing. 2018;24(3):134-41.
2. Swift MC, Scholten I. Not feeding, not coming home: parental experiences of infant feeding difficulties and family relationships in a neonatal unit. Journal of clinical nursing. 2010;19(1-2):249-58.
3. Wieczorkievicz AM, de Souza KV. O processo de amamentação de mulheres mães de crianças portadoras de síndrome de Down. Cogitare Enfermagem. 2009;14(3):420-7.
4. Donkor CM, Lee J, Lelijveld N, Adams M, Baltussen MM, Nyante GG, et al. Improving nutritional status of children with Cerebral palsy: a qualitative study of caregiver experiences and community-based training in Ghana. Food Sci Nutr. 2018;7(1):35-43.
5. Morrow A, Quine S, Craig J. Health professionals’ perceptions of feeding‐related quality of life in children with quadriplegic cerebral palsy. Child: care, health and development. 2007;33(5):529-38.
6. Barros da Silva R, Barbieri-Figueiredo MdC, Van Riper M. Breastfeeding Experiences of Mothers of Children with Down Syndrome. Comprehensive child and adolescent nursing. 2018:1-15.
7. Adams MS, Khan N, Begum S, Wirz S, Hesketh T, Pring T. Feeding difficulties in children with cerebral palsy: low‐cost caregiver training in Dhaka, Bangladesh. Child: care, health and development. 2012;38(6):878-88.
8. Zuurmond M, O'Banion D, Gladstone M, Carsamar S, Kerac M, Baltussen M, et al. Evaluating the impact of a community-based parent training programme for children with cerebral palsy in Ghana. PloS one. 2018;13(9):e0202096.
